# 75-year-old Woman with a Fever and Rash

**DOI:** 10.5811/cpcem.2019.7.44064

**Published:** 2019-07-22

**Authors:** Diane Kuhn, Jonathan Strong, Laura J. Bontempo, Zachary D.W. Dezman

**Affiliations:** * University of Maryland Medical Center, Department of Emergency Medicine, Baltimore, Maryland; † University of Maryland School of Medicine, Department of Emergency Medicine, Baltimore, Maryland

## CASE PRESENTATION (Resident Presentation)

A 75-year-old woman with a history of multiple myeloma presents to the emergency department (ED) with her daughter for chief complaints of fevers and a rash. Much of the history was provided by the patient’s daughter, her primary caregiver. The patient has had intermittent fevers for three days. The fevers occur once or twice a day, last several hours, with defervescence between episodes. The highest temperature recorded at home was an axillary temperature of 38.6 degrees Celsius (C). The patient developed a rash on her left wrist two days ago, and yesterday her lip became discolored. The patient reports that she has numbness and tingling on both her right and left forearms.

The patient was recently hospitalized for pneumonia. She was treated with intravenous (IV) antibiotics, after which the hospital team changed the patient to an oral antibiotic. The daughter believes the name of the oral antibiotic was “something with an ox.”

The patient was diagnosed with IgG lambda multiple myeloma in 2004 and treated with an autologous stem cell transplant in 2005. She has had multiple relapses but at the time of presentation was in partial remission. In addition, she has a history of therapy-related myelodysplastic syndrome, diabetes mellitus, hyperlipidemia, and stage III chronic kidney disease. Her home medications are daratumumab, bortezomib, lenalidomide, pomalidomide, dexamethasone, acyclovir, trimethoprim-sulfamethoxazole, azacitidine, glimepiride, insulin, simvastatin, magnesium, calcium carbonate, erythropoietin, and vitamin D. She is allergic to penicillins. She has a 30 pack-year smoking history but quit more than 25 years ago.

**Images 1A and 1B:**
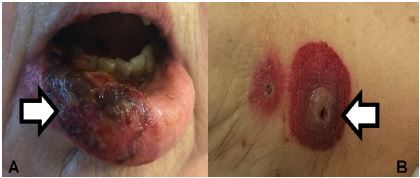
Purpura of the lip (A) and forearm lesion (B) of a woman with fever and rash.

Physical examination revealed an overweight (body mass index was 27) white woman sitting up in a stretcher. Her temperature was temperature 38.1°C, her blood pressure was 146/78 millimeters of mercury, her pulse was 86 beats per minute, and she was breathing 22 breaths per minute (bpm) with an oxygenation saturation of 95% on room air. Her head was atraumatic and normocephalic. Her extraocular movements were intact and her pupils were three millimeters in diameter, round, equal, and reactive to light. Her oral mucosal membranes were moist, with purpura of the lip (Image 1A). Her heart had a normal rate and regular rhythm without audible murmurs, rubs or gallops. She was mildly tachypneic without clear accessory muscle use. Her lungs had diffuse rhonchorous breath sounds bilaterally. She had an implanted vascular access port in her chest wall and the site was without erythema or induration. Her abdomen was soft with normal bowel sounds. She had a few small areas of ecchymoses over the abdomen. All four extremities were warm and well perfused. Her forearms were tender to palpation with some decreased sensation to light touch. She had a well circumscribed purpuric lesion on the left forearm as shown in Image 1B. Neurologically she was oriented to self, place, and time without cranial nerve deficits except decreased sensation in the right V3 region in the area of the purpuric lip lesion.

**Image 2:**
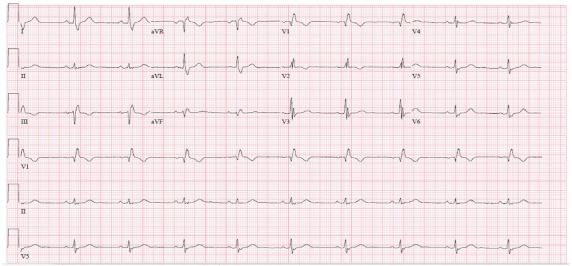
Electrocardiogram of febrile cancer patient showing sinus rhythm and known bifascicular block.

**Image 3:**
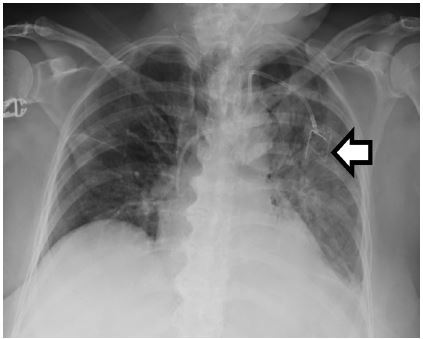
Chest radiograph of febrile cancer patient with left-sided chemotherapy port in place.

The patient’s initial laboratory results are shown in Table 1. These were significant for severe leukopenia, anemia, and thrombocytopenia consistent with known hematologic malignancy. Her chemistry showed mildly impaired renal function and elevated glucose. Table 2 shows the results of the cultures that were drawn from the patient in the ED. Her electrocardiogram (ECG) (Image 2) was unchanged from her baseline ECG. Her chest radiograph (CXR) is shown in Image 3. The patient received antipyretics and was started on broad-spectrum antibiotics before being admitted to the hospital for further workup. An additional test revealed the diagnosis.

## CASE DISCUSSION (Attending Discussion)

When presented with a complex case, I find it helpful to succinctly frame the patient presentation: I have a 75-year-old female with a past medical history of multiple myeloma and stem cell transplant, currently on a complex medication regimen including immunomodulators and chemotherapy, who presents with fever, pancytopenia (likely neutropenia), persistent respiratory symptoms despite antibiotics, and a rapidly progressive scattered purpuric and necrotic rash.

My immediate concern after reading this case is neutropenic fever, which is defined by a neutropenic patient experiencing a single oral temperature of ≥38.3^o^C or a temperature of ≥38.0^o^C sustained over a one-hour period.^1^ The description of the patient’s fever meets these criteria. Neutropenia is defined as an absolute neutrophil count (ANC) <1500 cells per microliter (μ/L).^2^ While the white blood cell (WBC) differential is not provided, the total WBC count is 1400 cells/(μ/L), so the patient has at least mild neutropenia. This places the patient at risk for a broad range of infectious organisms including opportunistic bacterial, fungal, and viral infections. Neutropenic patients may not develop symptoms of an infection due to a blunted immune and inflammatory response, leading to atypical presentations of infections. Per the American Society of Clinical Oncology and the Infectious Diseases Society of America guidelines, “in the absence of an alternative explanation, clinicians should assume that fever in a patient with neutropenia from cancer therapy is the result of an infection.”^3^

Now that the medical emergency has been addressed, we can examine the rash. Describing a rash using dermatologic terminology helps me narrow the differential diagnosis. This patient has several round, umbilicated purpuric papules and plaques on her left wrist and right forearm with central necrosis that appear in various stages of evolution. The distribution of the lesions is asymmetric and may correspond to where peripheral IV catheters were placed during her recent hospitalization. The patient also has a purpuric and necrotic rash on her right lower lip, with the purpura extending beyond the vermillion border. There appear to be pustules or a honey-colored / white crust present. Note that there is no mention of any lesions involving the oral mucosa. The physical exam describes small areas of ecchymoses on the abdomen. This may be part of the patient’s purpuric rash or heparin injections during her recent hospitalization. Now that I’ve described the rash, I will consider the broad categories of causes: bacterial, fungal, and viral infections, as well as non-infectious etiologies.

Bacterial infections are the most frequent cause of infection in patients with neutropenic fever.^3^ More than half are due to gram-positive organisms such as Staphylococcus or Streptococcus species (especially *Staphylococcus Epidermiditis*).^3^ She was recently hospitalized, raising suspicion for hospital-acquired and drug resistant organisms. I suspect the patient was discharged on levofloxacin (“something with an ox”), an antibiotic appropriate for treating community-acquired pneumonia and the empiric treatment of neutropenic fever.^1^,^3^ Purpura fulminans can occur in immunosuppressed patients and is described as petechiae that rapidly progress to purpura and necrotic lesions within hours. *Neisseria meningitidis* or varicella are the most common causes, but pneumococcus, Staphylococcus, and streptococci are also implicated. But patients with purpura fulminans are often critically ill, suffering from septic shock and disseminated intravascular coagulation, which does not fit with this patient’s presentation.

Our patient’s blood cultures did not grow any bacterial pathogens and her symptoms progressed despite antibiotics, so we have to consider fungal infections, especially since they can cause persistent or recurrent fevers. Our patient has a number of risk factors for fungal disease (a central venous catheter, hematologic malignancy, stem cell transplant, diabetes, and glucocorticoid treatment) and she is not on fluconazole prophylaxis.

Disseminated candidiasis can be seen in neutropenic patients after a disruption of the gastrointestinal mucosa (common in chemotherapy) or from central venous catheters. It presents as a scattered, diffuse, maculopapular or pustular rash; rarely the rash may be purpuric. Endophthalmitis may be seen, but lung involvement is rare, making this an atypical case and therefore disseminated candidiasis is unlikely in this patient.

Patients with disseminated aspergillus infections typically present with cough, fever, and hemoptysis. CXR may show single or multiple nodules with or without cavitation, but neutropenic patients may present with segmental consolidation as seen in our patient. Hematologic dissemination can rarely lead to skin lesions that typically manifest as subcutaneous nodules or pustules that evolve into purpuric and necrotic lesions with an ecthyma gangrenosum-like appearance.

Patients with diabetes and immunosupression are at risk for mucormycosis. The most common form is rhino-orbital-cerebral mucormycosis, which presents as erythema and swelling in the skin overlying the sinuses or orbits. The patient’s skin lesion is located on her lower lip, and she does not appear to have any sinus involvement making mucormyosis less likely. Cutaneous mucormycosis is rarer and is associated with trauma or wounds, appearing as a single painful ecythma-like lesion.

Endemic fungi such as *Histoplasma capsulatum*, *Blastomyces dermatiditis*, and *Coccidioides* spp are also possibilities. However, blastomyces and coccidioides are not endemic to the Mid-Atlantic, and Histoplasma is uncommon.^4^ The rash of disseminated histoplasmosis is described as diffusely scattered erythematous papules and nodules which may be umbilicated or crusted, inconsistent with this patient’s rash.

Reactivation of herpes simplex-1 (HSV-1), herpes simplex-2 (HSV-2), and varicella zoster virus are also important infections to consider in neutropenic patients or those taking medications such as bortezomib.^5^ However, the patient’s rash is not typical for herpes or zoster and the patient is taking acyclovir for prophylaxis.

Stevens-Johnson syndrome / toxic epidural necrolysis (SJS/TEN) is a non-infectious cause of rash that presents with fever and muco-cutaneous lesions. The rash typically begins on the face and trunk as coalescing macules with purpuric centers before spreading to the extremities in a symmetric distribution.^6^ Over hours to days bullae form, and the skin may begin to slough off. The oral mucosa and vermillion border are almost always involved, with hemorrhagic erosions covered by crusting or a grayish-white membrane. Patients with active malignancy are at increased risk and our patient is on multiple medications that have been implicated in SJS / TEN: lenalidomide, pomalidomide, and trimethoprim-sulfamethoxazole.^7^ However, the risk of SJS / TEN is thought to be limited to the first eight weeks of taking a medication. Further, our patient’s rash is not symmetric, making SJS / TEN less likely.

Thrombotic thrombocytopenic purpura (TTP) is worth considering. The classic pentad of TTP includes fever, thrombocytopenic purpura, microangiopathic hemolytic anemia, renal dysfunction, and neurologic symptoms. Our patient has fever, thrombocytopenic purpura, anemia, and renal dysfunction. The PLASMIC scoring system can be used to identify patients with severe ADAMS13 deficiency who would benefit from plasma exchange.^8^ While the patient has thrombocytopenia, her active cancer and her stem-cell transplant history, her low bilirubin, and high creatinine make it unlikely that she has TTP.

Multiple myeloma itself can very rarely present with fever and skin lesions. The skins lesions can occur due to infiltration of plasma cells into the skin (plasmacytomas), deposition of protein (amyloid, cryoglobulins), or secondary to cytopenias (i.e. thrombocytopenia). Plasmacytomas are typically not purpuric or necrotic, and the lesions do not arise over the course of hours to days. Amyloidosis secondary to multiple myeloma can present with purpuric lesions on the face and extremities, classically on the eyelids and periorbital area. The lesions often have a linear or geometric shape occurring due to minor trauma (“pinch purpura”). The patient’s lesions do not have this classic appearance or distribution. Further, the patient is also lacking other features of amyloidosis such as proteinuria and/or restrictive cardiomyopathy.

Cutaneous vasculitis can present with petechiae and purpura. If the vasculitis results in vascular occlusion the lesions can become necrotic and ulcerate. There is a long list of causes for cutaneous vasculitis including drug reactions, infections, malignancy, and rheumatologic causes. One potential cause of cutaneous vasculitis worth considering in this patient is cryoglobulinemia due to multiple myeloma. In cryoglobulinemia, cold causes high circulating levels of immunoglobulins to precipitate resulting in obstruction of distal small blood vessels and rarely an inflammatory vasculitis. Lesions have a predilection for the extremities, particularly acral areas with Raynaud phenomenon and digital ischemia sometimes occurring. Cryoglobulinemia is unlikely in this case given the distribution of the lesions, lack of reported cold intolerance, and lack of other organ involvement (i.e., glomerulonephritis).

One more non-infectious entity to consider are the neutrophilic dermatoses (ND) such as Sweet’s syndrome or pyoderma gangrenosum. There is an association between ND and hematologic malignancies, active chemotherapy, and being over the age of 65.^9^ In these conditions, inflammatory papules or pustules progress to ulcerations with a necrotic base and a bluish or violaceous margin. As in our case, the rash can present where a patient has had a recent procedure or injury. The diagnosis is made by meeting two minor criteria (recent respiratory illness, fevers, hematologic malignancy, recent vaccination, response to steroids, and elevated erythrocyte sedimentation rate) and two major criteria (classic rash and a biopsy showing neutrophilic infiltrate).^10^ While rare, this patient meets one of the major criteria and many of the minor criteria for ND. The one missing criterion is *a diagnostic test*.

If I were the first physician to care for this patient when they presented to the ED, my chief concern would be presumed sepsis in the neutropenic and febrile patient. Once the patient’s medical emergency is managed, I would consider other causes of their fever. In this case, I believe they have a ND, and a skin biopsy would be the diagnostic test of choice.

## CASE OUTCOME (Resident Presentation)

The diagnostic test ordered was a punch biopsy of the skin. The pathology report demonstrated sheets of neutrophils in the upper dermis with less involvement in the deeper dermis, consistent with acute neutrophilic dermatosis (Sweet’s syndrome). The leukemia service recommended starting the patient on one milligram per kilogram (mg/kg) of methylprednisone and the patient quickly defervesced. Steroids were tapered slowly over the following month with the lesions on the lip and upper extremities treated with local wound care. The patient was discharged shortly thereafter.

## RESIDENT DISCUSSION

Sweet’s syndrome was first described by Robert Sweet in 1964 as a constellation of symptoms including fever, leukocytosis, and tender erythematous papules and plaques, and a mature neutrophilic infiltrate.^11^ Sweet’s syndrome has since been linked to hematologic malignancies immune disorders, such as pyoderma gangrenosum, leading researchers to conclude that these diseases might be grouped as “a continuous pathologic spectrum**”** known as neutrophilic dermatoses ND.^12^

ND are characterized by a cutaneous neutrophilic infiltrate without evidence of infection. Patients with ND may have extracutaneous neutrophilic infiltrates, which will present as systemic diseases such as inflammatory bowel disease and rheumatoid arthritis. Traditionally, ND have transitional and overlap forms, or manifestations that either progress from one ND to another or represent an intersection of more than one ND.^12^

Sweet’s syndrome can be broken into classical, malignancy-associated, and drug-induced. Classical Sweet represents approximately half of all cases, typically affecting women 30–50 years old. The disease has no clear geographical or ethnic predisposition. The classical form most commonly involves the upper extremities and it may be preceded by a gastrointestinal or respiratory infection.

Fifteen to twenty percent of Sweet’s syndrome cases are associated with a malignancy, most commonly hematologic cancer**.**^13^ Acute myelogenous leukemia is the most common cancer, followed by myeloproliferative diseases such as that of our patient. Sweet’s syndrome can occur before, during, or after a malignancy, though the development of Sweet’s syndrome while in remission may signal disease recurrence.^1^

Drug-induced Sweet’s syndrome has been associated with immunomodulating drugs (granulocyte macrophage colony stimulating factor, filgrastim and lenograstim, ipilimumab, bortezomib, and azathioprine,) and chemotherapy agents.^14^ Surprisingly, azathioprine has been identified as both a possible cause and treatment for Sweet’s syndrome. Antibiotics (trimethoprim-sulfamethoxazole and minocycline), and anti-hypertensives (hydralazine) have been shown to induce Sweet’s syndrome. Our patient was taking three of these medications.

The pathophysiology of Sweet’s syndrome is uncertain. Marzano et al. (2014) performed protein arrays of patients with both pyoderma gangrenosum and Sweet syndrome, noting an increase in pro-inflammatory cytokines interleukin-1β, interleukin-6, and interleukin-8, and tumor necrosis factor-α.^15^ Increased levels of granulocyte-colony stimulating factor have also been associated with both acute myelogenous leukemia and Sweet’s syndrome.^16^ Recent research has found mutations in the isocitrate dehydrogenase protein, which activates oncogenes and inactivates tumor suppressor genes, in individuals with myelodysplastic syndrome and Sweet’s syndrome.^17^

The major criteria for classical or malignancy-associated Sweet’s syndrome include 1) an abrupt onset of painful erythematous plaques or nodules, and 2) histology of skin lesions showing a neutrophilic infiltrate in the absence of infection or vasculitis.^18^ Minor criteria include 1) fever greater than 38^o^C, 2) an association with malignancy, inflammatory or infectious disease, or vaccination, and 3) elevation of inflammatory markers such as erythrocyte sedimentation rate (ESR), C-reactive protein (CRP), white cell count or percentage of neutrophils. Patients with underlying hematologic malignancies may present with neutropenia rather than neutrophilia. Both major criteria and two out of four minor criteria are required for diagnosis. The diagnostic criteria of drug-induced Sweet’s syndrome also requires a temporal relationship between the offending agent and the course of symptoms.^19^ In the case presented, our patient met diagnostic clinical criteria and had features that were consistent with all three forms of Sweet’s syndrome.

The clinical course of Sweet’s syndrome is highly variable, with fever being the most common presenting symptom. Intermittent fevers may occur for days to weeks prior to development of cutaneous findings. Symmetric lesions on the upper extremities or head and neck are the most common cutaneous features. Although the cutaneous eruptions may begin as papules or nodules, they can enlarge and form into irregular plaques. It is not unusual for these lesions to occur in areas of cutaneous trauma, such as venipuncture sites. Patients often have hyperalgesia in the areas of skin findings, which resolves over the course of days to weeks with treatment.

Unfortunately, even characteristic clinical and historical disease features are inadequate to rule out other possible etiologies of fever, particularly in cancer patients who are immunocompromised. Malignancy-associated Sweet’s syndrome presents a unique diagnostic challenge as it is most commonly associated with hematologic cancers. Although the cyclic or intermittent fevers and dermatologic findings may be a diagnostic clue, a high level of suspicion must be maintained for other sources of fever. Thus, these patients should undergo a standard fever workup, including urinalysis, blood cultures, and CXR. In addition to complete blood count and chemistries of renal and liver function, inflammatory markers such as CRP and ESR may help to differentiate between sources of fever. Empiric antibiotic treatment while awaiting cultures is important in patients who present with febrile neutropenia of uncertain etiology. It is important to consider early involvement of dermatology for a skin biopsy in patients who have intermittent fevers and characteristic dermatologic findings.

First-line treatment of Sweet’s syndrome is 0.5–1.0 mg/kg continued for four to six weeks of systemic glucocorticoids.^20^ Symptoms generally improve or resolve within the first one to two weeks. Local corticosteroids may be used in cases of few lesions with no systemic symptoms. Potassium iodide has some efficacy and can be given as a 300 mg tablet three times daily or a solution dosed at 1000 mg/day. In the case of drug-induced Sweet’s syndrome, the offending agent should also be discontinued. There are literature reports of malignancy-associated Sweet’s syndrome resolving with treatment of the cancer.^19^,^20^ Similarly, an unknown malignancy must be considered when a diagnosis of Sweet’s syndrome is made.^20^

Since the episode, the patient has not had a recurrence of Sweet’s syndrome, although she has had progression of her multiple myeloma and myelodysplastic syndrome with poor prognosis.

## FINAL DIAGNOSIS

Sweet syndrome secondary to multiple myeloma and myelodysplastic syndrome.

## KEY TEACHING POINTS

- Consider non-infectious causes of fever in immunocompromised patients, particularly patients with known malignancies.
- Sweet’s syndrome should be considered in patients with a rash and fever, especially when there is associated malignancy, inflammatory or infectious disease, or vaccination.
- Chemotherapy patients are often on complicated regimens of medications with numerous and clinically relevant side effects; maintain a high degree of suspicion for the role of immunomodulators in disease processes.
- If an apparently healthy patient without recent medication changes or viral illness is diagnosed with Sweet’s syndrome, that patient should undergo a workup for underlying malignancy.
- Assume that neutropenic patients presenting with fever are septic and resuscitate them aggressively, even though they may appear clinically to be well.

## Figures and Tables

**Table 1:** Initial laboratory results for a 75-year-old woman presenting with fever and rash.

Complete blood cell count		Coagulation profile	
White blood cell count	1.4 K/μL	Prothrombin time	16.7 seconds
Hemoglobins	6.3 g/dL	Partial thromboplastin time	44.0 seconds
Hematocrit	19.10%	International normalized ratio	1.3
Platelets	14 K/μL	Urinalysis	
Serum chemistries		Appearance	Cloudy
Sodium	145 mmol/L	pH	5.0
Potassium	5.4 mmol/L	Ketones	Negative
Chloride	109 mmol/L	Bilirubin	Negative
Bicarbonate	24 mmol/L	Protein	Negative
Blood urea nitrogen (BUN)	91 mg/dL	Nitrite	Negative
Creatinine	2.1 mg/dL	Red blood cells	3–5 cells per high-powered field
Glucose	303 mg/dL	White blood cells	6–10 cells per high-powered field
Calcium	8 mg/dL	Venous blood gas	
Magnesium	2 mg/dL	FiO_2_	21%
Phosphorous	7 mg/dL	Respiratory rate	22 per minute
Total protein	4.7 g/dL	pH	7.39
Albumin	2.4 g/dL	pCO_2_	42 mmHg
Aspartate aminotransferase	63 μ/L	pO_2_	26 mmHg
Alanine aminotransferase	78 μ/L	HCO_3_	42 mEq/L
Alkaline phosphatase	206 μ/L	HBO_2_	43.70%
Total bilirubin	0.5 mg/dL	Base	−0.4 mmol/L

*FiO_2_,* fraction of inspired air; *pH,* potential of hydrogen; *pCO_2_,* partial pressure of carbon dioxide; *pO_2_,* partial pressure of oxygen; *HCO_3_,* bicarbonate; *HbO_2_,* oxyhemoglobin; *K/μL,* kilos per microliter; *mg/dL,* milligrams per deciliter; *g/dL,* grams per deciliter; *mEq/L,* milliequivalents per liter; *mmol/L,* millimoles per liter; *mmHg,* millimeters mercury.

**Table 2:** Culture results of woman with fever and rash.

Source	Result
Blood #1	No growth
Blood #2	No growth
Urine	No growth
Sputum	No growth
